# Exploring the influence of resiliency on physician trust in patients: An empirical study of Chinese incidents

**DOI:** 10.1371/journal.pone.0207394

**Published:** 2018-12-12

**Authors:** Jiangjie Sun, Liping Zhang, Ruochuan Sun, Yuanyuan Jiang, Xiuyun Chen, Chengsen He, Jiuchang Wei

**Affiliations:** 1 Health Management College, Anhui Medical University, Hefei, Anhui, China; 2 Clinical Medical College, Anhui Medical University, Hefei, Anhui, China; 3 Department of General Surgery, The First Affiliated Hospital of Anhui Medical University, Hefei, Anhui, China; 4 College of Nursing, Anhui Medical University, Hefei, Anhui, China; 5 University of Science and Technology of China, Hefei, Anhui, China; Medical University Graz, AUSTRIA

## Abstract

**Background:**

The tension in doctor-patient relationships is becoming progressively greater due to the high expectations of patients and the physicians’ work pressure. Recent studies have addressed factors which affect the tension of doctor-patient relationships, and our study continues this trend by looking at the influence of resiliency and physician trust in the patient (PTP), that is, how much the doctor trusts the patient.

**Methods:**

Based on a survey of 329 physicians, a descriptive analysis of measured data was done using SPSS 19.0. Pearson correlation coefficient analysis was used to examine the correlation between PTP and resilience and the demographic variables. KMO and Bartlett methods were used to examine the correlation between PTPS and resilience. The method of factor analysis was used for multicollinearity tests, and multiple stepwise regression analysis was used to explore the demographic factors correlated with PTP and resilience.

**Result:**

Our results indicate that the level of PTP is influenced by the age, education, and income of the doctors. Physician age and income are significantly and positively correlated with PTP, but education is significantly and negatively related. Age, education, and income also affect the level of psychological resilience of physicians. Resilience is positively correlated with age and education but is negatively related to income. Resilience positively influences PTP.

**Conclusion:**

The direct factors of PTP include resilience, age, education, and income, while gender, title, and hospital department were found to be indirect influencing factors. To meet goals expressed in Chinese government policy related to these issues, we suggest improving the level of education of the doctors, providing reasonable annual salary increases for doctors, easing the tensions involved in medical treatment, reducing the physicians’ work pressure, improving the physicians’ work environment, and enhancing the physicians’ professional sympathy. Through such measures, the level of PTP will be enhanced.

## Introduction

In China, the mutual trust within harmonious doctor-patient relationships has changed, and the ideal relationship of “health related and life entrusted” regarding medical professionals has suffered. Lack of trust between doctors and patients has become a reality, and this is the root cause of the deterioration of the doctor-patient relationship [1]. This trust crisis has become a hot topic in current social studies and on August 26, 2016, the Politburo Bureau of the Communist Party of China examined and approved the Outline of the Healthy China 2030 Plan. This plan clearly strengthens the humanistic care of medical services and is intended to build harmonious doctor-patient relationships.

The remainder of this article is organized as follows. Section 1 introduces the theory of physician trust in the patient (PTP) and resilience, and analyzes the relations of the two theories. Section 2 introduces the representativeness of sample sources in China, then discusses the Physician Trust in the Patient Scale (PTPS) and the Connor-Davidson Resilience Scale (CD-RISC). PTPS was used to measure the PTP. CD-RISC was used to measure the resilience of Chinese physicians. Section 3 reports the main results and is followed by a detailed discussion of the results in Section 4. Section 5 concludes the study.

### PTP and resilience

In the doctor-patient relationship, the patient is on the relatively weak side and so there are a large number of studies on the trust that patients have in their doctors. For example, Howe (2015) discusses how doctors can retain the trust of patients and families when they (the doctors) will not provide the treatment patients want [2]. Selected other papers are “The role of trust in doctor-patient relationship: Qualitative evaluation of online feedback from Polish patients” [3], “Trust in the doctor-patient relationship in developing healthcare settings: A quantitative exploration” [4], “Does being informed and feeling informed affect patients’ trust in their radiation oncologist?” [5], and “How do you measure trust in the health system? A systematic review of the literature” [6]. That the patient trusts the physician is understandable, but the converse, the physician trusting the patient, is also part of the doctor-patient relationship. This latter trust is called “physician trust in the patient” (PTP) and it can promote the patients’ trust in doctors. Conversely, if a doctor does not trust the patient, the patient will receive the doctor’s diagnosis and treatment negatively, which may affect the behavior of the patient. Therefore, it is necessary to research PTP. Recently, the People’s Daily newspaper published an article entitled “To cultivate the good root of trust in the doctor-patient relationship” [7], which publicly and strongly promoted the study of doctor-patient trust.

Resilience refers to an ability or trait that individuals exhibit when confronted with stress or adversity so that they can successfully adapt and develop well [8]. In recent years, risk events between doctors and patients have occurred frequently. Doctor-patient trust is complicated, and the doctors in China are burdened with heavy workloads, partly because the patients have high requirements for the doctors’ work. The contradiction between the growing health needs of the people and the scarcity of quality medical and health resources has become an unavoidable reality for Chinese society.

### The theoretical relationship between the PTP and resilience

Resilience explores the individual’s stress response from a new perspective, which is the dynamic process of individual protection and environmental risk. It plays a decisive role in individual stress response and can effectively reduce the risk of stress. A study found that people with high resilience have more positive emotions in the face of pressure [9]. It is also pointed out in other studies that high resilience individuals tend to have more stable, diverse, and powerful social support systems. They are good at using social support resources to reduce stressors and reduce social risk [10]. The Chinese domestic doctors’ profession has become a high-risk industry, due to factors such as diagnostic error, lack of nursing, lack of advanced technology, and the risk of new drug use. These and other factors may lead to anxiety among doctors, resulting in a low level of resilience. Oermann and Standfest (1997) point out that doctors may not be able to cope with the current levels of stress, which may affect their ability to work and their work confidence [11]. There is an analytic hierarchy model of resilience. The doctors with the lowest level of resilience only concern themselves with survival. They focus completely on protecting themselves through instinctive self-preservation against violence or emotional repression. Such doctors do not risk conflict in the face of patients and so they will not be able to talk about PTP. Doctors with resilience at the intermediate level have a defensive awareness and, when facing a doctor-patient conflict, they refuse to communicate with the patients to protect themselves. This attitude hinders the formation of PTP. Doctors with resilience at the advanced level are good at mobilizing social support resources, flexible handling of work pressure and depression, and communicating with patients in the face of possible medical risks. To improve the level of PTP and mitigate the tension aspect of the doctor-patient relationship, this study explored the relationship between PTP and resilience.

Based on the existing literature, we propose four hypotheses which relate to PTP, demographic characteristics, and the hospital department (HD). The following hypotheses are postulated.

Hypothesis 1: Resilience is positively correlated with PTP.
Hypothesis 2: A significant difference exists in PTP in different departments.
Hypothesis 3: Demographic characteristics of physicians (age, gender, education, income, title, and HD) are significantly related to resilience.
Hypothesis 4: Demographic characteristics of physicians (age, gender, education, income, title, and HD) are significantly related to PTP.

## Methods

### Sample and data collection

**Fig 1:**
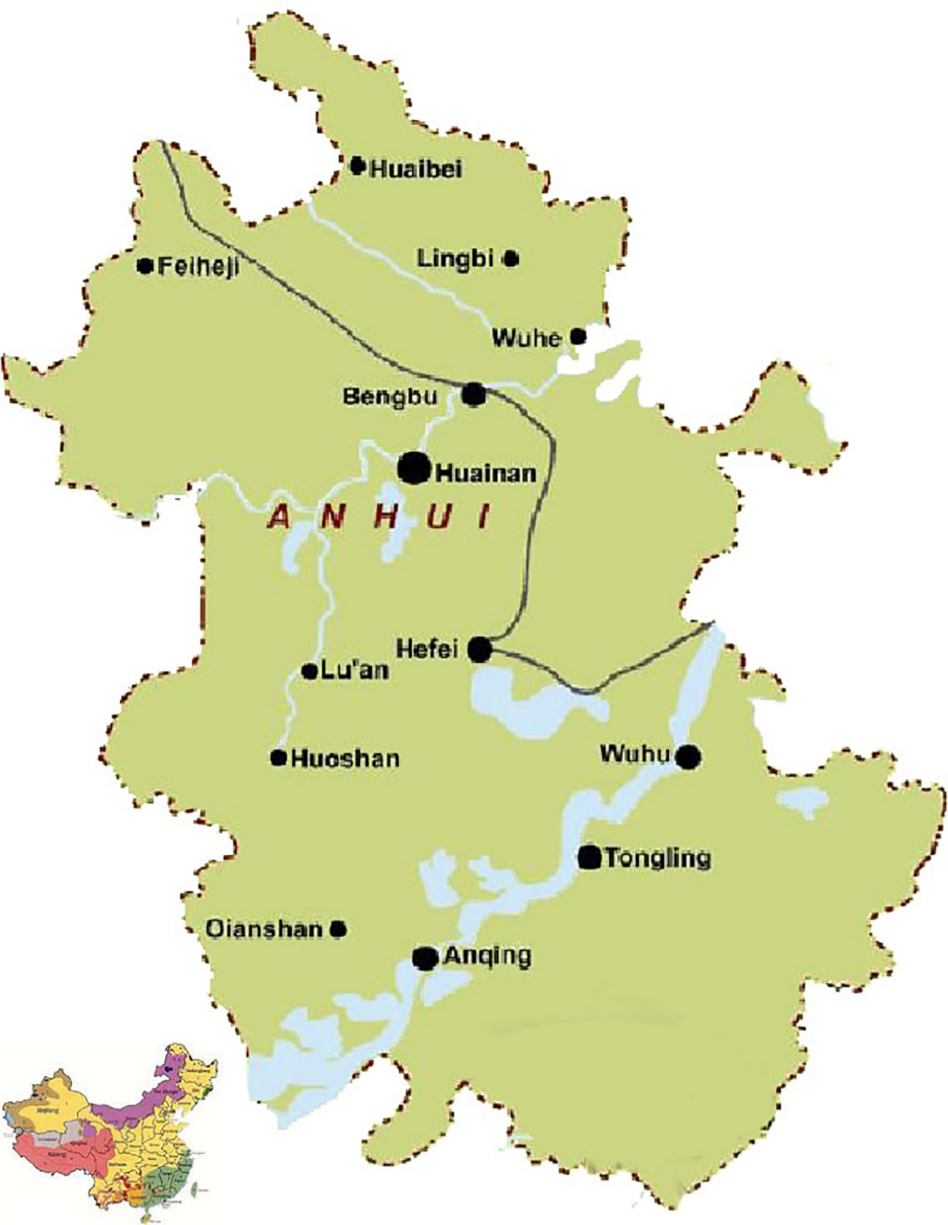
Map of anhui province in China.

The above hypotheses were tested using a survey of physicians of 10 general hospitals in Anhui, China. Anhui, Jiangsu, Shanghai, and Zhejiang together constitute the Yangtze River Delta urban agglomeration that has become one of the world’s six major world-class urban agglomerations. In 2014, Anhui won the title of “China’s most happy province” and was listed as the first experimental province of new urbanization in China (see Fig 1). It has more than 60 million residents.

**Fig 2:**
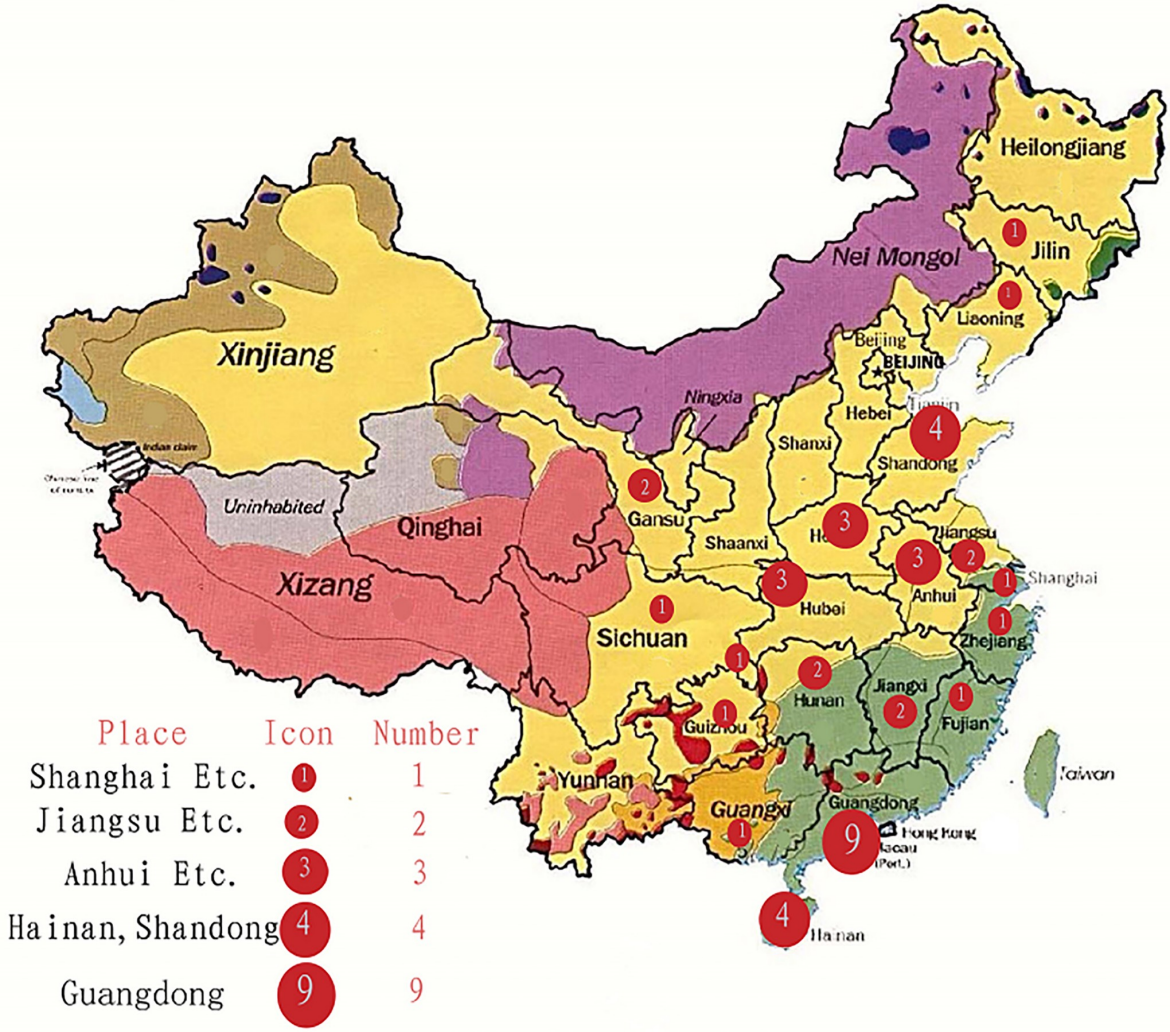
Quantity and distribution of physician/patient clashes in 2016.

It is worth discussing Hefei, the political, economic, and cultural capital of this landlocked central province. Identified by the Economist Intelligence Units as one of the top 20 emerging powerhouses in China [12], Hefei had the fastest growing metropolitan economy in the world in 2012 [13]. The medical and health undertakings in Anhui have been developing vigorously, and meanwhile, disputes between doctors and patients have occurred frequently. The hot events in the doctor-patient issues reported on the Internet show an increasing trend according to statistics in the People’s Daily Online Public Opinion Monitoring Room (as of December 14, 2016). In 2016, 43 cases of typical Chinese medical conflict happened in China, and the incidence rate of such clashes in Anhui was tied for fourth highest in China (see Fig 2). During that year, Anhui experienced three incidents which included two medical casualties (i.e., the deaths of two physicians) and a group incident which also had a bad influence. Therefore, the physicians of Anhui general hospitals are a suitable investigative sample for the study of resilience and PTP.

Ten comprehensive hospitals in Anhui province were selected for the study. The doctors surveyed included internists, surgeons, gynecologists, pediatric specialists, and those from other clinical departments. A total of 350 survey copies were issued and 329 valid questionnaires were recovered, so the response rate was 94%. Among the valid responses, 162 came from males and 167 from females. The subjects’ average age was 37.8 (SD = 11.2). For other demographic characteristics, see Table 1.

**Table 1:** Demographic characteristics of the subjects.

Variables		Number	Constituent ratio / %
Age / years			
	21~30	88	26.75
	31~40	108	32.83
	41~50	85	25.84
	>51	48	14.58
Education			
	Junior college	13	3.96
	College graduate	87	26.44
	Graduate/professional school	229	69.60
Title			
	Primary	95	28.88
	Intermediate	81	24.62
	Sub-senior	105	31.91
	Senior	48	14.59
HD			
	Internists	72	21.88
	Surgeon	72	21.88
	Gynecologist	62	18.84
	Pediatrics	64	19.45
	Other clinical departments	59	17.95
Income			
	<**$**10542.17	90	27.36
	**$**10542.17 to **$**18072.29	104	31.61
	>**$**18072.29	135	41.03

Notes: HD: Hospital department.

The survey was administered by researchers with a master of psychology degree and above: graduate students, psychology lecturers, and associate professors. This research team was trained before distributing the questionnaires. The questionnaire survey was conducted under the condition of informed consent of the respondents and a flash drive was given to them as a small thank-you gift after they completed the questionnaire.

Considering the heavy workload of doctors and the large number of participants, the authors of the study signed a document committing themselves to obtain the verbal informed consent of all survey respondents. This approval procedure was approved by the Ethics Committee of Anhui Medical University.

## Measures used

### PTPS

The study of doctor-patient trust began relatively early in the literature, and it mainly included research on the meaning and measurement of the trust between doctors and patients, using the idea that doctor-patient trust involves the patients’ expectation of the medical service provided by the doctors [14]. Trust is the pivot of the doctor-patient relationship [15]. Wallston first put forward the “nurse trusts patient scale” [16]. The measurement problem of nurses’ trust was solved, at least superficially, but using this scale, the reliability and validity were not given and the test-retest reliability Cronbach coefficient was only 0.32. Subsequently, a Stanford University professor set up the physician trust research group, revised the doctor-patient trust scale [17], and obtained the patient trust scale [18]. The study of PTPS introduced by Thom (2011) is the best basis for the patient scale of trust for this study [19] because this scale not only has high internal consistency (Cronbach coefficient 0.93) in Western countries but also has high internal consistency in China (Cronbach coefficient 0.92, P<0.001) [20]. In the current study, PTPS was used to measure the PTP by the Patient Role (Cronbach α coefficient 0.90, P<0.001) and Respect for Boundaries (Cronbach α coefficient 0.89, P<0.001).

Patient Role was measured by asking respondents to report their perception of PTP on a scale of “not at all confident (= 1); a little confident (= 2); somewhat confident (= 3); mostly confident (= 4); completely confident (= 5)” in relation to eight issues. These were “provides all the medical information you need”, “lets you know when there has been a major change in his or her condition”, “tells you about all medications and treatments he or she is using”, “understands what you tell him/her”, “follows the treatment plan you recommend”, “is actively involved in managing his/her condition/problem”, “tells you if he/she is not following the treatment plan”, and “keeps his or her appointments”.

Respect for Boundaries (RB) was measured by asking respondents to report their perception of PTP on a scale of “not at all confident (= 1); a little confident (= 2); somewhat confident (= 3); mostly confident (= 4); completely confident (= 5)” in relation to four issues, namely “respects your time”, “respects personal boundaries”, “does not make unreasonable demands”, and “does not manipulate the office visit for secondary gain” (e.g., for inappropriate disability certification or prescription of controlled substances).

The higher the score, the higher the level of the physician’s trust.

### CD-RISC

The resilience scale is a psychological measurement tool that was first developed in the study of post-traumatic stress disorder (PTSD) in the United States. The study of resilience has testified to the development of positive psychology. Resilience has been used to represent the individual’s capability of survival and adjustment after experiencing serious traumatic events [21]. Resilience is an ability or trait that an individual displays in the face of pressure or adversity, in order to adapt and develop successfully [22]. Tugade and Fredrickson argued that “psychological resilience refers to effective coping and adaptation although faced with loss, hardship, or adversity” [23]. Since 1993, several psychological instruments have proved to be reliable and valid to measure resilience [24–25] in the West. Among these instruments, the Connor-Davidson Resilience Scale [26–27] has earned much attention. Recent research has provided more evidence of the need for cross-cultural comparison of an imported construct and its measurement differences when applied in the West and East [26]. Yu and Zhang (2007) carried out exploratory factor analysis and confirmatory factor analysis using the Connor-Davidson Resilience Scale combined with data of the Chinese population and got the Chinese version of the CD-RISC [21]. This is the scale used in the current study to measure the resilience of Chinese physicians. The results have high reliability (Cronbach α coefficient 0.91, P<0.001) and are suitable for the study of doctors’ resilience in China.

The current study’s questionnaire measured resilience by tenacity (Cronbach α coefficients 0.88, P<0.001), strength (Cronbach α coefficients 0.80, P<0.001), and optimism (Cronbach α coefficients 0.60, P<0.001). The reliability coefficient of optimism was not as high as those for tenacity and strength, but it was acceptable in terms of the number of items it had (4 items only).

Tenacity describes an individual’s equanimity, promptness, perseverance, and sense of control when facing situations of hardship and challenge. Respondents were asked to report their state of tenacity in relation to thirteen issues: “prefer to take the lead in problem solving”; “when things look hopeless, I don’t give up”; “strong sense of purpose”; “think of self as strong person”; “in control of my life”; “I like challenge”; “one can achieve one’s goals”; “not easily discouraged by failure”; “under pressure, focus and think clearly”; “make unpopular or difficult decisions”; “can handle unpleasant feelings”; “know where to get help”; and “have to act on a hunch”.

Strength is described as an individual’s capacity of recovering and becoming strong after setbacks and past experiences. Respondents were asked to report their state of strength in relation to eight issues: “things happen for a reason”, “tend to bounce back after illness or hardship”, “best effort no matter what”, “able to adapt to change”, “coping with stress strengthens”, “past success gives confidence for new challenges”, “pride in your achievements”, and “one works to attain one’s goals”.

Optimism is described as an individual’s tendency to look on the positive sides of things and to trust one’s personal and social resources. Respondents were asked to report their state of optimism in relation to four issues: “see the humorous side of things”, “sometimes fate and God can help”, “close and secure relationships”, and “can deal with whatever comes”.

The participants were asked to respond on a 5-point Likert scale: “not true at all (= 1); a little true (= 2); somewhat true (= 3); mostly true (= 4); true all the time (= 5)”.

### Self-compiled questionnaire

The questionnaire included parts to gather demographic data about the subjects (gender, age, level of education, title, level of income) and hospital department (internist, surgical, gynecological, pediatric, and other clinical departments). The data was formatted as follows. Male (= 1), Female (= 2). Age was measured in years. Education was a three-point variable, namely junior college (= 1), college graduate (= 2), and graduate/professional school (= 3). Title was constructed with four categories, namely primary (= 1), intermediate (= 2), sub-senior (= 3), and senior (= 4). Income was a three-point variable, namely less than **$**10542.17 (= 1), **$**10542.17–**$**18072.29 (= 2), and over **$**18072.29 (= 3). The department data was specified as one of five categories, namely internist (= 1), surgeon (= 2), gynecologist (= 3), pediatric specialist (= 4), and other clinical departments (= 5)

### Statistical methodology

Two graduate students reviewed all questionnaire data, and EpiData 3.1 was used for data entry. Using SPSS 19.0, a descriptive analysis was done for the measurement data (mean, standard deviation). Pearson correlation coefficient analysis was used to examine the correlation between PTP and resilience and the demographic variables. KMO and Bartlett methods were used to examine the correlation between PTPS and Patient Role and RB. KMO and Bartlett methods were also used to examine the correlation between resilience and tenacity, as well as strength and optimism. The method of factor analysis was used for multicollinearity tests and multiple stepwise regression analysis was used to explore the demographic factors of PTP and resilience. Differences are considered to be statistically significant when P<0.05.

## Results

According to the guidelines proposed by Comrey, a sample size of 100 is too small, whereas 200 is passable, 300 is excellent, 500 is good, and 1000 is very good [28–29]. For the general analysis of the following 40 item factors, in most cases, 200 samples are sufficient. Tinsley suggested that the subjects ratio be five to ten [30]. The sample size of our study was 329, and the subjects ratio of PTPS and CD-RISC was 27.42 and 13.16 respectively, indicating that the amount of research data applied in the study met these criteria. Based on the sample data of PTPS and CD-RISC, we created a binary variable correlation matrix for each dimension and demographic data distribution (Table 2).

**Table 2:** Correlation matrix of the dimensions of PTPS, CD-RISC, and demographic variables.

Variables	Mean	SD	1	2	3	4	5	6	7	8	9	10	11
1. Age	38.76	11.19	1										
2. Gender	1.51	0.50	0.03	1									
3. Education	2.66	0.55	‑0.16	-0.18	1								
4. Income	2.32	1.04	0.67	0.11	0.11	1							
5. Title	2.14	0.82	0.85	0.24	0.39	0.76	1						
6. HD	2.90	1.41	0.15	-0.01	-0.14	0.05	0.10	1					
7. PR	27.14	5.75	0.45	0.12	-0.22	0.31	0.38	0.04	1				
8. RB	9.39	3.81	0.31	‑0.43	0.06	0.05	0.23	0.04	0.65	1			
9. Tenacity	49.22	7.06	0.12	0.33	0.13	‑0.27	0.15	0.09	0.65	0.58	1		
10. Strength	31.37	3.86	0.12	0.38	0.06	0.33	0.24	0.07	0.53	0.39	0.77	1	
11. Optimism	12.98	2.37	‑0.19	-0.08	0.07	‑0.14	‑0.31	0.13	0.35	0.52	0.61	0.46	1

Notes: HD:Hospital department; PR:Patient Role; RB:Respect for Boundaries; All r>.10 significant at p < .05. All the significant correlations are highlighted.

Table 2 shows the descriptive statistics of the PTPS, CD-RISC, and demographic variables, and gives the correlation of the bi-variable.

The shadow correlation in Table 2 was statistically significant. Generally, there will be a multicollinearity problem when the correlation coefficient is more than 0.9, and there may be a problem when the correlation coefficient is over 0.8, so 0.6 is the baseline for an acceptable correlation coefficient. Multiple collinearity tests were performed considering the patient role and RB as two dimensions of the PTP scale, and tenacity, strength, and optimism as three dimensions of the CD-RISC [31]. We adopted KMO and Bartlett methods to examine the correlation between PTP, patient role, and RB (KMO = 0.50, P = 0.000). KMO and Bartlett were also used to examine the correlation between resilience and tenacity, as well as strength and optimism (KMO = 0.64, P = 0.000). The results indicate that the PTP correlations with patient role and RB are significant. The experimental results indicate that tenacity, strength, and optimism are all significantly correlated with resilience.

We conducted Pearson correlation analysis on CD-RISC & PTPS (r = 0.66, P = 0.000), and the results supported Hypothesis 1: Resilience is positively correlated with PTP.

Considering the demographic variables, the correlation coefficients were all over 0.6 and multiple collinearity tests were conducted. Often, the larger the variance inflation factor (VIF), the greater the problem of multicollinearity. More concretely, multicollinearity is not a problem if the tolerance value is greater than 0.10 or the variance inflation factors (VIFs) are less than 10 [13,32]. In our study, the lowest tolerance value is 0.45 and the highest VIF is 2.22. Accordingly, multicollinearity does not appear to be a significant problem in our dataset.

The regression model for PTPS and CD-RISC on demographic characteristic variables is provided in Table 3.

**Table 3:** Prediction of PTPS and CD-RISC.

Predictor	Step 1		Step 2	
	β/*Coef*	*SE*	β/*Coef*	*SE*
PTPS				
Age	5.19***	0.88	4.88***	0.60
Gender	1.40	0.87		
Education	-2.42**	0.86	-2.41**	0.83
Income	1.47*	0.74	1.83*	0.74
Title	-0.53	0.92		
HD	-0.94	0.31		
Adjust R^2^	0.204		0.205	
F	15.034***		29.110***	
CD-RISC				
Age	6.13***	1.24	5.88***	0.85
Gender	1.99	1.23		
Education	4.67**	1.21	4.51***	1.67
Income	-2.82**	1.17	-3.34**	1.05
Title	-0.66	1.30		
HD	0.43	0.44		
Adjust R^2^	0.144		0.142	
F	10.20***		19.104***	

Notes: PTPS: Physician trust in the patient Scale; CD-RISC: Connor-Davidson Resilience Scale; HD:Hospital department

*

**, and

*** indicate P<0.05, P<0.01, and P<0.001, respectively.

Some of the findings in Tables 2 and 3 confirm Hypotheses 3 and 4. Demographic characteristics (age, education, and income of the physician) are significantly related to resilience, but resilience is uncorrelated to gender, title, and HD.

Our results partially give support for Hypothesis 4. Demographic characteristics (age, education, and income) are significantly related to PTP, but PTP is not related to gender, title, and HD.

The results show that PTPS was not significantly correlated with the department, so Hypothesis 2 was not confirmed.

The results of Tables 2 and 3 also show significant correlations between education and gender, title and age, professional title and income, hospital department and gender, and hospital department and education.

## Discussion

The results of this study show that the age of the physician, the degree of education, and the degree of the annual income affect the level of the PTP. PTP is positively correlated with the age and the income of the physician but negatively related to the degree of education. One of the possible reasons is that older doctors are more experienced in medical practice, and the more skillful they are in analyzing medical problems, the more favorable their positions and the higher the level of trust between such doctors and their patients. These results are consistent with the findings of Sun et al. [33]. Increased income can provide doctors with a good living environment, stimulate doctors’ positivity, and make doctors more confident in handling the doctor-patient relationship. According to the Table 2 data analysis results, possible reasons for the connection to title can be obtained. The physician title is positively related to the doctor’s annual income and physician age, which is consistent with the Chinese physician career promotion mechanism and the Chinese hospital management system. For Chinese physicians, title promotion requires attaining a certain number of working years and at present, in most hospitals in China, a doctor’s annual income is linked with title (the higher the title, the higher the annual income). Possible reasons for the education factor are that highly educated physicians, who are mostly on the front lines of medical work, have heavy work tasks and pressure, but in recent years, frequent medical disputes, the disgracing of doctors, and other uncivilized phenomena have not been uncommon. Some media report such phenomena in a biased manner, and hospitals pay compensation for economic losses to achieve the purpose of silence. This adds some mental pressure to medical staff, dampens the medical staff’s working enthusiasm, and makes the medical staff have a negative mood about current doctor-patient relationships. This also corresponds to the view of Lo Coco et al. that “frustrating experiences are a significant cause of negative emotional induction” [34]. In addition, some of the highly educated doctors have become the backbone of the hospital. The existence of overconfidence leads to a lack of communication at work which easily leads them to take a self-centered view of doctor-patient disputes, and this negatively affects the level of PTP [33]. Of course, there are other reasons also.

In this study, the age, level of education, and annual income of the physician were found to influence the psychological resilience of the physician. The psychological resilience of the physician is positively correlated with age and education degree but negatively correlated with annual income. One of the possible reasons is that the ability to handle emergencies is greater in the physician who is older. Highly educated physicians and those with more abundant experience also have higher resilience levels because they handle emergencies well. These results are consistent with the findings of Wagnild [35].

The negative correlation between income and resilience may result from the fact that, under the Chinese domestic performance salary system, the higher the doctor’s income, the heavier the workload they have. High-income physicians are employed in jobs which are often difficult, such as surgeons who often do life-saving surgery, and the amount of surgery performed by surgeons is relatively large every day. Therefore, there are frequent opportunities for dissension between doctors and patients; the psychological trauma of doctors is inevitable and poor psychological resilience is understandable.

The negative correlation between resiliency and income may also stem from higher-income physicians bearing more responsibility to handle stressful events, such as when new viruses require new drugs or technologies to treat them, which happens frequently. Due to the lack of doctors’ knowledge of new drugs and technologies to control the new virus, the incidents caused by a new virus will increase exponentially for a fairly long time [36]. The risk of failure to control such viruses will increase doctors’ frustration and thus reduce their psychological resilience [37–38]. We conducted Pearson correlation analysis on doctors ‘ annual income and HD (r = -0.01, P>0.05), as shown in Table 2. From the actual annual income data, we can see that surgeon income is relatively high, a phenomenon which may also be related to China’s performance salary system.

Another factor to consider when looking at resiliency is that some unscrupulous doctors lie about the presence of medical phenomenon in order to improve their income through treating imaginary illnesses. This fraud leads to patients to bear excessive medical expenses and the medical staff service attitude is not good [39–40], which means that patients (rightly) do not trust the physician. Long-term pressure or adversity in the physician’s Psychological Contract Game erodes tenacity. The positive power of physicians is conquered by a desire for money and the optimistic attitude of all participants naturally disappears, so the negative influence on the level of psychological resilience among such physicians is understandable. This is one of several possible reasons for a reduction of resilience.

In this study, the education level of male subjects was higher than that of female subjects, and the reason for this is thought to be that even in the 20^th^ century, the Chinese culture’s traditional gender roles exert a great influence. A possible reason for the existence of the correlation between department and gender lies in the practices of the departments of gynecology and surgery: In the Chinese cultural context, female doctors are considered to be more suitable for medical issues specific to women and so female gynecologists are common. On the other hand, surgical practice frequently requires much energy by the surgeons and male physical fitness is typically higher than female, so males dominate in Chinese surgery departments.

The results of this study show that resilience is an important protective factor for PTP. This may be due to the same theoretical properties just discussed because resilience is a positive psychological phenomenon. At the same time, PTP comes mainly from the physicians’ individual physical and mental factors, so we can use PTPS as a tool to study the positive performance of physician trust perception. PTP requires security, a sense of belonging, love, and a sense of value; the satisfaction of these needs depends on the internal environment (such as income) and external environment (such as the public opinion of doctor-patient disputes). At the same time, changes in the working environment are not automatically transferred into the individual’s consciousness. The concept of resilience emphasizes the identity construction of the individual in the social environment so that one’s sense of self-satisfaction is not affected by changes in the social environment. Therefore, we are led to the conclusion that resilience is an important protective factor for PTP.

## Conclusion

In this study, Pearson correlation coefficient analysis was used to analyze the correlation between PTP, psychological resilience, and certain demographic variables. Multiple stepwise regression analysis was used to explore the demographic factors correlated to PTP and resilience.

The results of this study show that the resilience of doctors positively affects PTP. The higher the resilience of the physician, the easier it is for that doctor to adapt to the temporary changes of work tasks, and the higher the ability to be calm in the face of setbacks and adversities. Resilient physicians can successfully respond to and maintain the normal functions in times of emergency and during negative events [41]. From a fundamental realization of the patient as the center, respecting interpersonal relationships provides a good foundation for PTP, and in such conditions a high level of PTP is inevitable. Qualitative conclusions were obtained by Krot and RudawskaI in the form of online research in Poland [3], where it was found that PTP positively influenced the relationship between doctors and patients. The authors also believe that providing good job education and promoting the resilience of doctors is the only way to improve the level of PTP and thereby mend the current tense doctor-patient relationship in China.

These results indicate that resilience, age, education, and annual income of the physician are direct factors that influence PTP, and gender, title, and department are indirect factors that influence PTP. To establish the “health related and life entrusted” relationship advocated in Chinese government policy goals, it is necessary to enhance the physician’s educational level, improve the doctor’s annual salary scientifically and reasonably, relieve the tension in the lives of medical doctors, reduce the doctors’ working pressure, enhance the professional identity of doctors, and improve the PTP level.

The results of this study indicate that the level of PTP could be improved by using a resilience adjustment model reasonably [42]. Such practices could include carrying out lifelong education in Medical Professionalism, strengthening the sense of honor and achievement of licensed doctors, enhancing the positive emotion involved in medical work, and relieving or eliminating some causes of negative emotions among doctors. In this way, society can improve the level of doctors’ resilience, weaken the negative impact of doctor-patient risk, and consequently reach a high level of PTP.

The method used in this study was a questionnaire filled in by physicians, which entails two limitations. The first is information bias. This study does not consider information such as the doctors’ marital status, the number of children they have, and the local GDP per capita, yet all of these factors are related to the doctors’ personal economic situations and can be used as indicators of their life stressors. Although including more such factors in this study may potentially provide a more comprehensive understanding of an individual’s economic status, more factors should add to the findings. In fact, the focus of our study was that demographic factors ARE correlated to resilience and PTP, the authors believe it may not affect the results and findings of this study. The second limitation of this study stems from response bias. The doctors may not be able to provide their real answers to some questions at the stage of data collection. To reduce the impact of response bias on our study, we grouped the demographic characteristics during the design process of the research. Further research could include an expansion of the sample size, the use of comparison and control methods, and the provision of dynamic management information which might provide a better understanding of the PTP predictions revealed in this paper.

## Supporting information

S1 File Physician Trust in the Patient Scale (PTPS). (PDF)

S2 File Connor-Davidson Resilience Scale (CD-RISC). (PDF)
