# Supplementary material for: Exploring the influence of resiliency on physician trust in patients: An empirical study of Chinese incidents
Source: PLoS One. 2018 Dec 12;13(12):e0207394. doi: 10.1371/journal.pone.0207394 (PMC6291099; doi:10.1371/journal.pone.0207394)
Supplement: S1 File — (PDF) [file pone.0207394.s001.pdf]

Physician Trust in the Patient Scale (PTPS)

The following is an optional topic,Tick the option you agree with to indicate your level of agreement or disagreement,among them “not at all confident (=1); a little confident (=2); somewhat confident (=3); mostly confident (=4); completely confident (=5)”

Ordin	Items	Not at all confiden ⟷ Completely confident				
1	Provides all the medical information you need	1	2	3	4	5
2	Lets you know when there has been a major change in his or her condition	1	2	3	4	5
3	Tells you about all medications and treatments he or she is using	1	2	3	4	5
4	Understands what you tell him/her	1	2	3	4	5
5	Follows the treatment plan you recommend	1	2	3	4	5
6	Is actively involved in managing his/her	1	2	3	4	5
7	Tells you if he/she is not following the treatment plan	1	2	3	4	5
8	Keeps his or her appointments	1	2	3	4	5
9	Respects your time	1	2	3	4	5
10	Respects personal boundaries	1	2	3	4	5
11	Does not make unreasonable demands	1	2	3	4	5
12	Does not manipulate the office visit	1	2	3	4	5
