# Supplementary material for: Exploring the influence of resiliency on physician trust in patients: An empirical study of Chinese incidents
Source: PLoS One. 2018 Dec 12;13(12):e0207394. doi: 10.1371/journal.pone.0207394 (PMC6291099; doi:10.1371/journal.pone.0207394)
Supplement: S2 File — (PDF) [file pone.0207394.s002.pdf]

Connor-Davidson Resilience Scale (CD-RISC)

The following is an optional topic,Tick the option you agree with to indicate your level of agreement or disagreement,among them “not true at all (=1); a little true (=2); somewhat true (=3); mostly true (=4); true all the time (=5)”.

Ordin	Items	Not true at all ⟷ True all the time				
1	Prefer to take the lead in problem solving	1	2	3	4	5
2	When things look hopeless, I don’t give up condition	1	2	3	4	5
3	Strong sense of purpose	1	2	3	4	5
4	Think of self as strong person	1	2	3	4	5
5	In control of my life	1	2	3	4	5
6	I like challenge	1	2	3	4	5
7	One can achieve one’s goals	1	2	3	4	5
8	Not easily discouraged by failure	1	2	3	4	5
9	Under pressure, focus and think clearly	1	2	3	4	5
10	Make unpopular or difficult decisions	1	2	3	4	5
11	Can handle unpleasant feelings	1	2	3	4	5
12	Know where to get help	1	2	3	4	5
13	Have to act on a hunch	1	2	3	4	5
14	Things happen for a reason	1	2	3	4	5
15	Tend to bounce back after illness or hardship	1	2	3	4	5
16	Best effort no matter what	1	2	3	4	5
17	Able to adapt to change	1	2	3	4	5
18	Coping with stress strengthens	1	2	3	4	5
19	Past success gives confidence for new challenges	1	2	3	4	5
20	Pride in your achievements	1	2	3	4	5
21	One works to attain one’s goals	1	2	3	4	5
22	See the humorous side of things	1	2	3	4	5
23	Sometimes fate and God can help	1	2	3	4	5
24	Close and secure relationships	1	2	3	4	5
25	Can deal with whatever comes	1	2	3	4	5
